# Success stories in genomic medicine from resource-limited countries

**DOI:** 10.1186/s40246-015-0033-3

**Published:** 2015-06-18

**Authors:** Konstantinos Mitropoulos, Hayat Al Jaibeji, Diego A. Forero, Paul Laissue, Ambroise Wonkam, Catalina Lopez-Correa, Zahurin Mohamed, Wasun Chantratita, Ming Ta Michael Lee, Adrian Llerena, Angela Brand, Bassam R. Ali, George P. Patrinos

**Affiliations:** The Golden Helix Foundation, London, UK; University of Maastricht, Maastricht, The Netherlands; Department of Pathology, College of Medicine and Health Sciences, United Arab Emirates University, Al-Ain, United Arab Emirates; Laboratory of NeuroPsychiatric Genetics, Biomedical Sciences Research Group, School of Medicine, Universidad Antonio Nariño, Bogotá, Colombia; Unidad de Genética, Grupo GENIUROS, Escuela de Medicina y Ciencias de la Salud, Universidad del Rosario, Bogotá, Colombia; Division of Human Genetics, Faculty of Health Sciences, University of Cape Town, Cape Town, South Africa; Genome Quebec, Montreal, Canada; Department of Pharmacology, Faculty of Medicine, University of Malaya, Kuala Lumpur, Malaysia; Department of Pathology, Medical Genomic Center, Ramathibodi Hospital, Faculty of Medicine, Mahidol University, Bangkok, Thailand; Laboratory for International Alliance on Genomic Research, RIKEN Center for Integrative Medical Sciences, Yokohama, Japan; Institute of Biomedical Sciences, Academia Sinica, Taipei, Taiwan; CICAB Clinical Research Center, Extremadura University Hospital and Medical School, Badajoz, Spain; Department of Pharmacy, University of Patras School of Health Sciences, Patras, Greece

**Keywords:** Genomic medicine, Pharmacogenomics, Economic evaluation in pharmacogenomics, Genome informatics, Public health genomics, Genetics education, Africa, Latin America, Southeast Asia

## Abstract

In recent years, the translation of genomic discoveries into mainstream medical practice and public health has gained momentum, facilitated by the advent of new technologies. However, there are often major discrepancies in the pace of implementation of genomic medicine between developed and developing/resource-limited countries. The main reason does not only lie in the limitation of resources but also in the slow pace of adoption of the new findings and the poor understanding of the potential that this new discipline offers to rationalize medical diagnosis and treatment. Here, we present and critically discuss examples from the successful implementation of genomic medicine in resource-limited countries, focusing on pharmacogenomics, genome informatics, and public health genomics, emphasizing in the latter case genomic education, stakeholder analysis, and economics in pharmacogenomics. These examples can be considered as model cases and be readily replicated for the wide implementation of pharmacogenomics and genomic medicine in other resource-limited environments.

## Introduction

Advances in genomics and related technologies have revolutionized the practice of medicine by means of better diagnosing and/or prognosing human inherited disorders and cancer and rationalizing drug use [1]. Translation of genomic findings into health care and health systems has been catalyzed by the advent of genome-wide studies, in which next-generation sequencing for either targeted, whole-exome, and/or whole-genome sequence analyses stands as the most powerful approach [2]. These approaches are gradually being adopted by diagnostic laboratories and hospitals in the USA and Western Europe, with several tangible results in the context of the implementation of genomic medicine. In addition, there are several guidelines both from the US Food and Drug Administration (FDA; http://www.fda.gov) and the European Medicines Agency (EMA; http://www.ema.europa.eu) regarding the use of genome-based therapies.

However, the pace of implementation of genomic medicine practices outlined above is not always equally met in developing and resource-limited countries, where significant barriers exist, often related to lack of tangible resources as well as technology and knowledge transfer. Furthermore, biomedical scientists and health care professionals in these countries frequently fail to fully appreciate the potential that this new approach offers to improve medical diagnosis and treatment. As such and taking into consideration that approximately 85 % of the world’s population lives in developing/resource-limited countries, making genomic medicine accessible in these countries represents a major opportunity and challenge. There have been recently some significant examples from the successful implementation of genomic medicine in resource-limited settings in Europe and Asia, which could stand as examples for replication in similar environments, towards a broader implementation of genomic medicine and pharmacogenomics.

### Translating genomics into health care of resource-limited countries

Successful translation of genomics into genomic medicine relies on several related disciplines, such as population genomics, pharmacogenomics, or informatics and the approach/task of public health genomics, which are frequently intersected. Below, we describe some examples from successful implementation of genomic medicine related to these disciplines and approaches.

### Development of a European-wide pharmacogenomics map: the Euro-PGx project

The implementation rate of pharmacogenomics in the various health systems in Europe is very heterogeneous. This is not only due to the variable degree of harmonization of the national guidelines within Europe, particularly related to health care and education, but also most importantly due to differences in resource availability, making implementation of pharmacogenomics in resource-limited European countries an even more challenging task [3]. Also, there is very little knowledge regarding the pharmacogenomic biomarker allele frequencies in various European countries, to shortlist the different actionable pharmacogenomic biomarkers that would be useful in clinical settings of different countries, on which to base drug dose recommendations. The Euro-PGx project (http://www.goldenhelix.org/index.php/research/pharmacogenomics-in-europe) aims to determine the varying pharmacogenomic biomarkers allele frequencies in a large number of mostly developing European countries in an effort to produce drug dose recommendations for different countries. Preliminary findings from a large-scale genotyping effort using the DMET+ microarray (Affymetrix, Santa Clara, CA, USA), including 1936 pharmacogenomic biomarkers in 231 pharmacogenes, suggest that the allele frequencies of several pharmacogenomic biomarkers vary significantly among different European countries, despite the fact that the vast majority of the European populations have a very strong Caucasian genetic component. In particular, as far as anticoagulation treatment is concerned, significant differences were found in allele frequencies of the CYP2C9*3 allele in the Serbian population, while there are differences in the average warfarin dosing in the different European countries investigated (Mizzi et al., in preparation). These varying frequencies in the pharmacogenomic biomarkers in different European populations will be made accessible to the scientific community through the FINDbase database (http://www.findbase.org) using the microattribution approach [4]. Also, in an effort to increase pharmacogenomics education and awareness among health care professionals, the Euro-PGx project coordinates the organization of pharmacogenomics educational activities in various European countries, namely the Golden Helix Pharmacogenomics Days (http://pharmacogenomicsdays.goldenhelix.org). Until June 2015, 15 different events have been organized in ten European, mostly developing, countries, with the expectation to expand these activities in other developing nations around the world.

### Pharmacogenomics in clinical settings

Perhaps one of the most characteristic and noteworthy examples of success story in pharmacogenomics is the discovery of the association of the HLA-B*1502 allele with the devastating carbamazepine-induced Stevens-Johnson Syndrome/Toxic Epidermal Necrolysis (SJS/TEN) [5] and of the HLA-B*5801 allele with allopurinol-induced severe cutaneous adverse reactions [6] in Southeast Asia and particularly in Taiwan. Subsequently, a large clinical study conducted in Taiwan confirmed the benefit of HLA-B*1502 testing to prevent carbamazepine-induced SJS-TEN by using HLA-B*1502 screening to prospectively identify subjects at genetic risk for the condition [7]. This led the Taiwanese government to cover and reimburse the costs for HLA-B*1502 genetic testing since 2010.

From the numerous pharmacogenomics studies conducted to date, a large number of genomic biomarkers have been shown to be correlated with variable drug efficacy and/or toxicity. However, only a small fraction of these drugs have been approved by regulatory agencies to bear pharmacogenomic information in their labels. To facilitate exploitation of this valuable information from clinicians, who often lack the necessary genomics education, it is of utmost importance to make this knowledge readily available in a format that can be easily digested by clinicians. To fulfill this requirement, DruGeVar database (http://drugevar.genomicmedicinealliance.org) [8] was developed, representing an online knowledge portal for clinical pharmacogenomics which triangulates drugs with genes and pharmacogenomic biomarkers. This online resource includes those pharmacogenomic biomarkers that have been approved by regulatory agencies and allows, through a user-friendly data querying and visualization interface, formulation of queries that would guide clinicians to order the correct genetic test prior to prescribe a drug to a patient and by this, providing an individual theranostic service.

In addition, in order to record patients’ pharmacogenomic biomarkers, a pharmacogenomic card has been proposed in Southeast Asia and the Ramathibodi Hospital in Thailand has launched a “pharmacogenomics” wallet card, aiming to summarize patients’ HLA gene variant information predicting risk of developing SJS/TEN from specific drugs, a devastating and often fatal cutaneous adverse reaction [9]. Such pharmacogenomics card would be readily expandable to more drug/pharmacogenomic biomarkers to serve patients’ needs, depending on the medication that they receive.

In Latin America, the Iberian American Network of Pharmacogenetics and Pharmacogenomics (RIBEF, created in 2006) aims to promote collaborative pharmacogenomics research in Iberoamerica. RIBEF currently consists of 43 research groups and more than 200 researchers with the main goals and principles to promote scientific studies among its members and clinical implementation of pharmacogenomics to improve drug efficacy and security with the ultimate goal of helping the health-care needs of neglected populations. As such, the aims of the Network are to develop human resources training, to produce research and to disseminate information in universities and hospitals, and to promote clinical implementation of pharmacogenomics. Up to date, RIBEF teaching programs and human resources training activities include over 400 events all over Latin America. Also, another goal of the RIBEF network is to develop research projects, among which are population pharmacogenomics studies of Iberoamerican populations. For that purpose, the CEIBA Consortium (Consorcio Europeo e Iberoamericano de Farmacogenética de Poblaciones) was established among the RIBEF members (Ibero-Spain and Portugal and Latino American Network of Pharmacogenomics) to study population pharmacogenomics of these populations. The MESTIFAR project aimed at determining variability of polymorphisms in genes involved in response to drugs in populations of different ethnic origin [Native Americans (Amerindian) and Mestizos (the result of post-Columbian admixture)]. In total, more than 6000 healthy volunteers have been evaluated, constituting one of the largest populations pharmacogenomics study conducted today. Besides population pharmacogenomics, the network has other ongoing projects that are related to clinical pharmacogenomics in neurology, psychiatry, cardiovascular and infectious diseases, etc., with up to a total of 31 scientific articles being published so far.

In Africa, there is a disproportionate burden of disease with the triple challenge caused by HIV/AIDS, tuberculosis, and malaria against a backdrop of an increasing burden of non-communicable diseases [10]. Genomic analyses have found that several genetic variants can provide increased resistance or susceptibility to HIV infections [11]. Moreover, more than 80 % of therapeutic drugs used in the management of these diseases/conditions are metabolized by cytochrome P450 (CYP) enzymes that exhibit genetic polymorphisms. Recent studies have provided evidence of a huge variability in the pattern of genetic variations in the CYP genes among African populations that was translated into differences in drug response [12].

Lastly, in the Middle East, pharmacogenomics research started in 1996 in the United Arab Emirates (UAE), initially involving erythrocyte glucose-6-phosphate dehydrogenase deficiency (G-6-PD) related with drug-induced hemolytic anemia [13] and later arylamine N-acetyltransferase (NAT2) [14]. Also, the *CYP2D6* pharmacogenomic marker allele frequencies were investigated in the Emirati population, including reporting of four novel *CYP2D6* variants [15], while a warfarin pharmacogenomics study is currently under way for the Emirati population [16] (AlJaibeji and coworkers; unpublished). These studies sparked an interest from Dubai Hospital to integrate pharmacogenomics information for chemotherapeutic agents, while the UAE Health Authority policy of reporting adverse drug reaction in the UAE requires expert pharmacogenomics recommendation within the first 24 h related to each adverse drug reactions reported [17].

### Mapping of stakeholders in genomic medicine

A plethora of key players and stakeholders compose the genomic medicine puzzle, whose level of genomics awareness and views varies significantly. Systematic mapping of the views and opinions of the various stakeholders on genomic medicine would positively impact on better understanding the corresponding policy environment and the role of the relevant key stakeholders in the field, on identifying the main opportunities and obstacles for evidence-informed policy-making and timely implementation in pharmacogenomics and genomic medicine, and on prioritizing the various stakeholders’ needs, in an effort to better plan the undertaking of various measures in genome-based health care, i.e., from the perspective of public health genomics.

Recently, Mitropoulou and coworkers undertook such initiative to assess the level of support or opposition to pharmacogenomics and genomic medicine in Greece [18]. This survey indicated that the majority of the key stakeholders, namely academic institutions and research organizations, the bioethics council, private genetic laboratories, citizens, pharmaceutical and biotechnology companies, genetics and genomics professional associations, private health insurance, industry, pharmacists, and physicians (both geneticists and other specialties), are highly supportive of pharmacogenomics and genomic medicine in Greece. On the contrary, the Ministry of Health and the social health insurance funds oppose to the implementation of genomic medicine, while the Greek National Medicines Organization displays a neutral stance, possibly since the cost-effectiveness and cost-benefit of a pharmacogenomics approach is not yet fully proven, proper legislation to oversee the operation of private genetic testing laboratories is not yet in place, or simply because they fear that reimbursement of genetic testing could increase rather than decrease the overall health-care expenditure. These latter stakeholders have high intervention power against the implementation of pharmacogenomics and genomic medicine into mainstream clinical practice. Subsequently, several opportunities and obstacles in the pharmacogenomics and genomic medicine policy-making in Greece are derived from this analysis, based on the current position and intervention potential of the key stakeholders. Similar analysis is currently on its way in Middle Eastern countries to determine the current position and views of the corresponding stakeholders in these countries.

Also, insufficient genomics education and lack of genomics awareness among health-care professionals and the general public are two perspectives of the same issue, which hinders the smooth incorporation of genomic medicine into clinical practice [19]. On the one hand, the vast majority of health-care professionals state that they feel insufficiently trained in genomics to be able to engage with the delivery of genome-based services [18], while on the other hand, patients and the broader public tend to have low genomic literacy, which impairs their capacity to successfully integrate genome-based information into their personal decision-making, which is a challenge for public health genomics and here especially for the field of health literacy [20]. On top of this, genomics education is not uniformly provided in various academic institutions worldwide, with the USA and Western European countries to lead the way and the Southeastern European countries lagging behind. A recent survey in 175 departments from 98 universities from 11 Southeastern European countries indicated that for a significant number of universities, the topic of pharmacogenetics/pharmacogenomics is not included at all in their undergraduate and postgraduate curricula in health sciences [21]. Also, studies surveyed Greek and Italian physicians indicated that only a small fraction of those feel competent enough either to propose a genetic test for their patients and/or to interpret the results from such a test [21, 22]. These findings are in sharp contrast with the current reality of pharmacogenomics education in northern European countries, where pharmacogenomics are more uniformly and extensively taught and highlight the need for a more in-depth genomics education, either with the incorporation of pharmacogenomics and genomic medicine in their undergraduate or graduate training, or in the form of continuous medical education seminars. These studies might provide the basis to harmonize pharmacogenomics education in southeast European countries with those of northwest European countries, such that it would directly impact on a smoother and timely integration of pharmacogenomics into mainstream medical practice.

As in other resource-limited regions, in Latin America there are very few postgraduate programs focusing on genomics [23]. Finally, in Africa, the high cost of genomic services and low private investment is compounded by a relatively low level of medical professionals with understanding of genomics [24]. In addition, a recent attempt in sub-Saharan Africa to triangulate the views of multiple stakeholders related to sickle-cell disease (SCD), namely doctors, parents with SCD-affected children, and adult SCD patients towards prenatal diagnosis of SCD, showed several discrepancies. The majority accepted the principle of prenatal genetic diagnosis for SCD (78.7, 89.8, and 89.2 %, respectively); however, parents (62.5 %) were more in favor of termination of SCD-affected pregnancy, than doctors and adults patients (36.1 and 40.9 % acceptance, respectively). These differential attitudes signal potential value-based conflicts on the horizon and can usefully inform the future policy actions in the African continent, as OMICS biotechnologies are increasingly employed in global health [25]. Specially, there are encouraging evidence of participation of African-based scientists in studying the genomics of monogenic diseases [26–29] and multifactorial conditions [30]. These data are concretely assisting the effective practice of genomic medicine, that is well established in South Africa [31], in some Northern African countries [32, 33], and recently initiated in Central Africa [34]. In addition, regional initiatives such as the launch of the Southern Africa Human Genome Project by the South African government [35] have been boosted by international funding agencies and academic institutions, through major programs such as the Malaria Genomic Epidemiology Network (MalariaGEN) (http://www.malariagen.net/), the Human Heredity and Health in Africa (H3Africa) program [36], and the African Genome Variation Project [37]. The fortunate association of international consortia efforts will enhance the regional and local initiatives to build the capacity in research skills and overcome the barriers for the use of genomics to address the disease burden of Africans.

Current populations in Latin America are characterized by high and heterogeneous levels of admixture, arising from the history of these regions and corresponding to different patterns of mating between individuals of Native American, European, and African descent [38]. As an interesting international example, the National Institute of Genomic Medicine was built in Mexico with public funds [39], with several publications in the fields of population genomics and medical genomics [40, 41]. Other Latin American countries, such as Brazil and Colombia, have had success in the implementation of genetic and genomic approaches for the study of several human diseases with high epidemiological impact in those resource-limited countries [42–45], while an equally important element that is helping to implement genomics in these countries is the availability of commercial tests, by service providers abroad.

### Next-generation sequencing and genomic medicine

The next-generation sequencing (NGS) technology, which was introduced 8 years ago, marked the beginning of a new era concerning the analysis of human genome sequences [46–49]. Before the advent of NGS, Sanger sequencing was widely used for screening variants potentially causing monogenic and complex diseases which were located in coding and regulatory genomic regions. However, studies involving numerous genes or large genomic regions were particularly challenging due to intrinsic technical limitations (e.g., read length encompassing up to 700 base-pairs (bp) per reaction). On the contrary, the NGS approach allows simultaneously analyzing millions of bp in only hours, thereby facilitating large-scale exploration of the human genome [1]. The first NGS studies and many projects nowadays have been focused on researching novel recessive disease-related sequence variants, particularly those caused by homozygous mutations. Such variants are relatively easy to identify because most are novel and/or are exclusively present in several members of the same family. This allows easy screening of candidate variants in public SNP databases and filtering them between affected and non-affected individuals from the same family. Successful screening attempts have also been described for monogenic dominant Mendelian disorders. However, this approach implies more drawbacks because heterozygous mutations (logically) occur more frequently, thereby involving complex filtering to select potentially deleterious mutations. NGS has not been widely used for some complex pathologies in which several variants might contribute towards the phenotype, because data analysis highlights remarkable complexity, especially for simultaneous interactive network exploration.

Three main approaches are normally used at present. They mainly depend on the length of the genome region being analyzed: whole-genome sequencing (WGS), whole-exome sequencing (WES), and custom target sequencing microarrays (TSM) [1]. WGS is mainly used for research purposes while WES and TSM are used for both research and diagnosis. Most academic and private technological platforms for the above are located in high-income countries. This might be due to the high cost of NGS technology, such as new-generation sequencers and bioinformatics platforms. However, resource-limited countries have performed interesting studies by using NGS outsourcing services. For instance, using such NGS services in Colombia has led to identifying novel genes and mutations causing monogenic diseases [43, 44, 50]. Furthermore, innovative diagnostic approaches have been proposed for pathologies having overlapping phenotypes and which are caused by various genes [43, 44]. The available data regarding the genomes of particular mammalian species has enabled large-scale comparative genomics approaches leading to dissecting loci related to evolution mechanisms and potentially contributing towards human diseases [51]. Moreover, complex pathologies such as female infertility have also been explored via NGS (Fonseca et al. in press).

### Economic evaluation in pharmacogenomics

Considering the fact that genome-based drug treatment can contribute towards the reduction of national health-care expenditures, mostly by reducing hospitalization due to the various adverse drug reactions, this need is more than ever urgent, particularly in resource-limited countries, which in many cases have vast fiscal deficits [52].

Although the field of economic evaluation in genomic medicine, pharmacogenomics, and public health genomics is currently in its infancy, the majority of the economic evaluation studies indicate that genotype-guided therapy can be cost-effective and of high cost-benefit in several countries. Particularly related to resource-limited countries, initial economic evaluation studies in the Thai population indicated that HLA-B*15:02-guided carbamazepine treatment is cost-effective in Thailand compared to conventional treatment and can reduce the carbamazepine-induced severe adverse drug reactions [53, 54]. As a result, the Thai government is now providing HLA-B*15:02 testing as a standard of care, while the same findings were reported for the Singaporean population, when cost-effectiveness of HLA-B*1502 genotyping in adult patients with newly diagnosed epilepsy was assessed [55].

Similarly, a recent study to evaluate cost-effectiveness of warfarin treatment in Croatian elderly ischemic stroke patients with atrial fibrillation indicated that 97.07 over 89.12 % of patients belonging to the pharmacogenomics-based and the control group, respectively, did not present major complications, while the incremental cost-effectiveness ratio of the pharmacogenomics-based versus the control groups was estimated at €31,225/quality-adjusted life year (QALY), indicating that pharmacogenomics-guided warfarin treatment may represent a cost-effective therapy option for the management of elderly patients with atrial fibrillation who developed ischemic stroke in Croatia [56].

## Conclusions and future perspectives

**Fig. 1:**
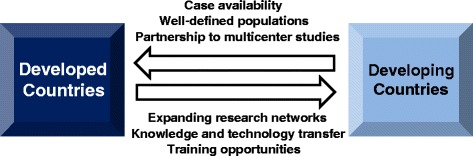
Encouraging collaboration between developed and developing/resource-limited countries in the field of genomic medicine. While developing countries will benefit from training opportunities, knowledge transfer, and expanding research networks, developed countries are also likely to benefit through comparative work and multicenter projects involving cases with rare diseases or unique clinical features from well-defined populations

Implementation of genomic medicine in resource-limited environments will be only made possible though stronger collaboration in genomics research between developed and developing/resource-limited countries, which is likely to create benefits for all parties. To this end, developing countries will undoubtedly benefit from training opportunities, knowledge transfer, and expanding transnational networks, while developed countries are likely to benefit through comparative work and multicenter projects on families with rare diseases or unique clinical features (Fig. 1) [57].

The outcomes of the studies, outlined in the previous paragraphs, indicate that, as far as genomic medicine is concerned, developing countries may be resource-limited but are also potential-rich in producing data in various genomic medicine-related disciplines, from the perspective of public health genomics. Although this summary of examples depicting successful implementation of genomic medicine in resource-limited countries is far from complete, it sets a paradigm for replication in other countries, contributing towards not only acquiring more and better insights into the requirements to implement genomic medicine in these environments but also harmonizing the strategies and policies for the faster and smoother adoption of genomic medicine practices in the various national health-care systems.
